# Intrafractional motion models based on principal components in Magnetic Resonance guided prostate radiotherapy

**DOI:** 10.1016/j.phro.2021.09.004

**Published:** 2021-10-04

**Authors:** Samuel Fransson, David Tilly, Anders Ahnesjö, Tufve Nyholm, Robin Strand

**Affiliations:** a Department of Surgical Sciences, Uppsala University, Uppsala, Sweden; b Department of Medical Physics, Akademiska Hospital, Uppsala, Sweden; c Elekta Instruments AB, Stockholm, Sweden; d Department of Immunology, Genetics and Pathology, Uppsala University, Uppsala, Sweden; e Department of Radiation Sciences, Umeå University, Umeå, Sweden; f Department of Information Technology, Uppsala University, Uppsala, Sweden

**Keywords:** Principal component analysis, Intrafractional motion, Prostate cancer

## Abstract

**Background and purpose:**

Devices that combine an MR-scanner with a Linac for radiotherapy, referred to as MR-Linac systems, introduce the possibility to acquire high resolution images prior and during treatment. Hence, there is a possibility to acquire individualised learning sets for motion models for each fraction and the construction of intrafractional motion models. We investigated the feasibility for a principal component analysis (PCA) based, intrafractional motion model of the male pelvic region.

**Materials and methods:**

4D-scans of nine healthy male volunteers were utilized, FOV covering the entire pelvic region including prostate, bladder and rectum with manual segmentation of each organ at each time frame. Deformable image registration with an optical flow algorithm was performed for each subject with the first time frame as reference. PCA was performed on a subset of the resulting displacement vector fields to construct individualised motion models evaluated on the remaining fields.

**Results:**

The registration algorithm produced accurate registration result, in general DICE overlap >0.95 across all time frames. Cumulative variance of the eigen values from the PCA showed that 50% or more of the motion is explained in the first component for all subjects. However, the size and direction for the components differed between subjects. Adding more than two components did not improve the accuracy significantly and the model was able to explain motion down to about 1 mm.

**Conclusions:**

An individualised intrafractional male pelvic motion model is feasible. Geometric accuracy was about 1 mm based on 1–2 principal components.

## 1 Introduction

For radiotherapy of prostate cancer the uncertainties in target position is normally handled by assigning a margin around the clinical target volume (CTV) to form a planning target volume (PTV) to which the dose is prescribed. The van Herk [1] recipe is commonly applied in obtaining the margin with input based on population statistics of the target position uncertainties. However, since the planned distribution rarely conforms exactly to the PTV it has been shown to overestimate the margin required [2]. It is also only strictly applicable if the treatment consist of sufficiently many fractions to support its inherent assumption of uncertainty distribution convolutions which breaks down in the case of hypofractionation [3]. Here the dose blurring due to random deviations, i.e. intrafractional motion and uncertainties in setup, over the whole treatment period can no longer be assumed to populate the underlying probability distributions enough to motivate simple convolutions to derive the end result. In the light of the prolonged treatment time for hypofractionated treatments, as well as potential of a dose painting approach [4], the demand for accurate delivery of the radiation increases and with that requirements for intrafractional surveillance.

The introduction of MR-Linac systems combining an MR-scanner with a Linac for radiotherapy enables imaging both prior to treatment and during treatment with high contrast for soft tissue. Hence there is a possibility to acquire individualised learning sets for motion models prior to each fraction. Motion models through principal component analysis (PCA) have been suggested to account for periodic motion such as respiration [5], [6], [7]. PCA has also been applied in interfractional modeling of organ deformations in the pelvic region and applications in treatment simulation [8], [9], [10], [11]. The potential for dimensionality reduction to describe the motion pattern with only one or two components is appealing [12]. A lot of attention has been given to model respiratory intrafractional motion [13] and little or none for other types of motion. It is hence natural to investigate if there is a potential benefit in constructing intrafractional models of pelvic motion, with associated bladder and rectum motion and the resulting prostate move. Such a model could be utilized to drive e.g. a real-time adaptive approach, similar to [12], or potentially construct patient specific margins for both the target and organs at risk.

Or aim with this work was to construct PCA-based intrafractional motion models from MR-data of the male pelvic region and evaluate the accuracy of such models. The outlined workflow was described and discussed in the context of MR-Linac, however could be modified to fit a more general context, e.g. cone beam CT-based workflows.

## 2 Materials and methods

For prostate radiotherapy (RT) the region of interest is the lower pelvic containing the organs prostate, bladder and rectum. We aimed for construction of a joint model for these organs to simultaneously describe motions of these organs. The model requires a dataset for extraction of motion patterns, hence multi-time frame (dynamic) datasets are required. The motion was deduced from deformable image registrations (DIR) of each of the time frame in dynamic datasets, and the displacement vector fields (DVF) obtained from the registrations were broken down into their corresponding principal components. The registration accuracy is hence of great importance and requires attention prior going into construction of the model. The model itself was then validated. The data was hence divided into two cohorts, a training data set with the first *k* time frames and a validation set with the remaining time frames.

### 2.1 Image data

Dynamic scans of nine healthy male volunteers were utilized for the study with age span of 25–63 years (with approval from the Swedish Ethical Review Authority). Each subject was scanned during a period of 10–15 min with a dynamic 4D-sequence, and with the field of view covering the whole pelvis to avoid potential fold over artifacts, resulting in ten time frames for each subject. The scan parameters are given in Table 1 in supplementary material. A radial blade acquisition was utilized, oversampling the center of the k-space region to reduce motion sensitivity, e.g. breathing, and to achieve good contrast with reasonable scan time. The sequence was T2-weighted to get an adequate soft tissue contrast. Prior to registration manual segmentation of the organs of interest, i.e. prostate (target) and bladder and rectum (organs at risk) was performed by a single observer.

### 2.2 Image Registration

Image registration within each dynamic series was performed to obtain the DVFs for the model. An optical flow algorithm, suitable for the relatively small motion, was utilized [14] with a multiscale approach (available in MICE toolkit, NONPI Medical AB) to get the DVFs for each time frame relative to the reference here taken as the first time frame. Three pyramid levels and downsampling factor of 2 was utilized. The registration was stopped at a downsampling of 2 from the original resolution for a smooth displacement vector field. The image registration was based on the image intensities only.

The quality of the registration was evaluated using the DICE coefficient, measuring the overlap between binary images, here the reference (first time frame) image segmentation and the remaining time frames segmentations which were deformed using the DVFs obtained from image registration. The average hausdorff distance for the segmentation edges was also obtained with respect to the reference segmentation.

Inverse consistency [15] as vector magnitude error (VME) was evaluated to verify that the algorithm produced reasonable deformations. The registration direction was inverted and the forward and inverse transform composed and summed voxelwise

(1) $$\mathrm{VME}=\frac{1}{N}\overset{N}{\underset{r=1}{\sum }}|T_{I_{1}\to I_{2}}\circ T_{I_{2}\to I_{1}}(r)|$$

where $I_{1}$ and $I_{2}$ are the images, *N* the number of voxels and $T_{I_{1}\to I_{2}}$ and $T_{I_{2}\to I_{1}}$ the transforms between the frames of reference. The evaluation was performed only in the voxels belonging to the segmented regions.

### 2.3 Construction of the motion model

The motion model was constructed through PCA of the DVFs obtained from the deformable image registration. The PCA decomposes the DVFs into orthonormal components, resulting in a set of principal components, PCs, and their corresponding eigenvalues which describe the variance of each component, i.e. the amount of variability of the motion the components explain. If the components are ordered in descending eigenvalue order the first components accounts for the most of the variance in the modelled data. The PCs can be weighted and combined to describe the motion pattern. The procedure is straightforward and described elsewhere, e.g. [5], [8].

New states were generated through a weighted sum of the components to produce new fields,

(2) $$x_{\mathrm{model}}=\overset{\sim }{x}+\overset{k}{\underset{n=1}{\sum }}w_{n}{\mathrm{PC}}_{n}\;\|{\mathrm{PC}}_{n}\|=1$$

where $x_{\mathrm{model}}$ is the new DVF, $\overset{\sim }{x}$ is the centered displacement, $w_{n}$ the weight for the *n*:th principal component, ${\mathrm{PC}}_{n}$ the *n*:th principal component and *k* the number of principal components utilized. The PCs were normalized with the total length of all constituent vectors in the component. For a given displacement field x the optimal weight for each component is the projection of the component onto the centered displacement field, i.e.

(3) $$w_{\mathrm{opt},n}=(x-\overset{\sim }{x})\cdot {\mathrm{PC}}_{n}$$

We chose the first five DVFs for the training data and the remaining consecutive four DVFs for model validation data. From the training data different models were constructed utilizing part of the training data or all, in order to evaluate if results will change with the different amount of training data. The PCA was only performed on the boundary of the segmentations since the motion can be captured at these voxels, also reducing the influence of the potential chaotic behaviour of the motion captured by the image registration inside e.g. the bladder.

### 2.4 Model Validation and Evaluation

As a measure of the performance of the model the average geometric residual was utilized. This is the residual displacement not handled by the model and is simply calculated as

(4) $${∊}_{i}=\sqrt[{}]{\|x_{\mathrm{model}}-x_{\mathrm{ref}}{\|}_{i}^{2}}$$

where ${∊}_{i}$ is the residual for the *i*:th voxel in the model belonging to the segmentation boundary, $x_{\mathrm{model}}$ and $x_{\mathrm{ref}}$ are the model and the reference DVFs at that voxel, respectively. $x_{\mathrm{model}}$ is taken from Eq. 2 with the optimal weight from Eq. 3. This residual was obtained for each voxel in the model and averaged to get the average residual.

The resulting model obtained DVFs was also inverted and applied to the fixed image segmentation to generate a new volume. The method of Chen et al. [16] was chosen for the inversion step. The overlap between the fixed segmentation and the model generated was measured through the DICE coefficient.

## 3 Results

### 3.1 Image Registration

**Fig. 1:**
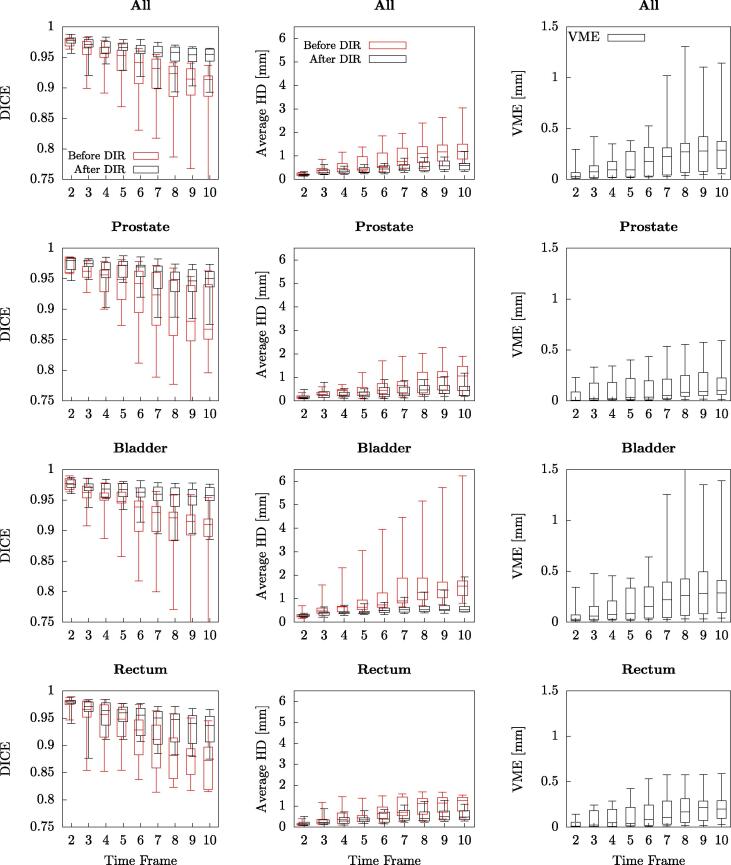
Results before and after deformable registration (DIR) for all subjects. Results are divided into time frames with one box for each time frame where the whiskers represent the maximum and minimum values. Red boxes are values before registration and black after for the left column. The registration was evaluated both on the joint segmentation for all organs as well as for each of the included organs separately. To the left the registration evaluation measure was the DICE overlap (boxes slightly horizontally shifted for a clearer presentation), in the middle average hausdorff distance and to the right the inverse consistency expressed as Vector Magnitude Error.

The results from the deformable image registration, Fig. 1, showed a general consistent registration accuracy for each time frame with a DICE coefficient over 0.95. One subject deviated slightly due to a larger motion pattern through a larger degree of bladder filling during the scanning, which in turn affected the prostate motion.

### 3.2 Model Validation and Evaluation

**Fig. 2:**
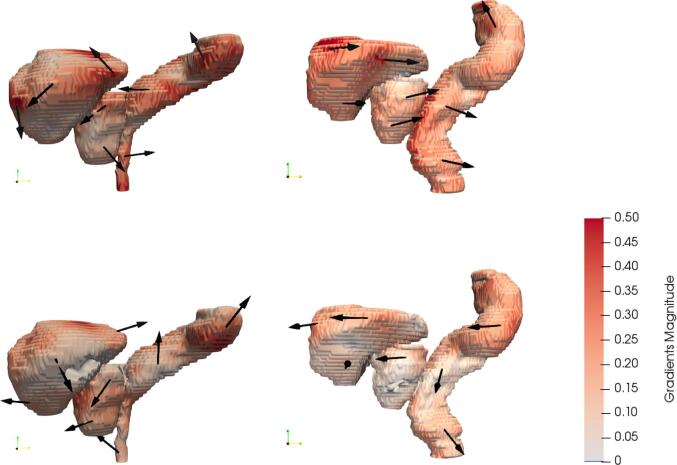
Example of the first (top) and the second (bottom) principal component for two of the subjects. The direction of each component is represented with the arrows and the size with the coloring of the surface (darker means a larger motion), where the size is the motion of one standard deviation.

A varying size and direction of the motion between subjects resulted in different motion models for each subject, exemplified in Fig. 2.

**Fig. 3:**
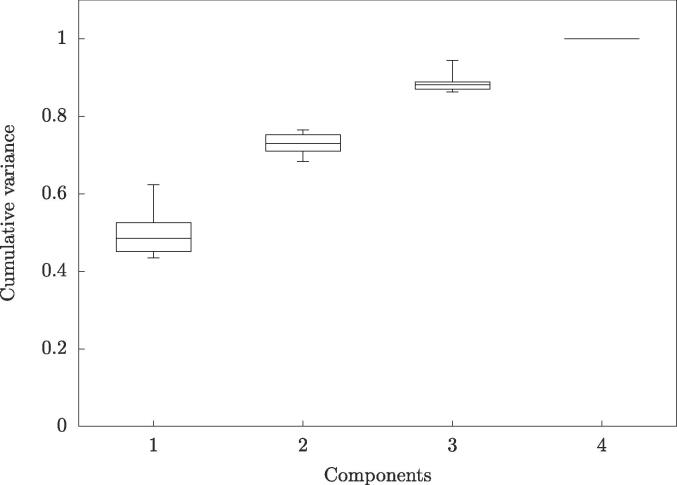
The cumulative variance for all subjects represented as boxplots for each number of components where the whiskers represent maximum and minimum values.

The cumulative variance, Fig. 3, shows that about 50% of the motion in the training data was explained in the first principal component, here with a model based on 5 DVFs.

**Fig. 4:**
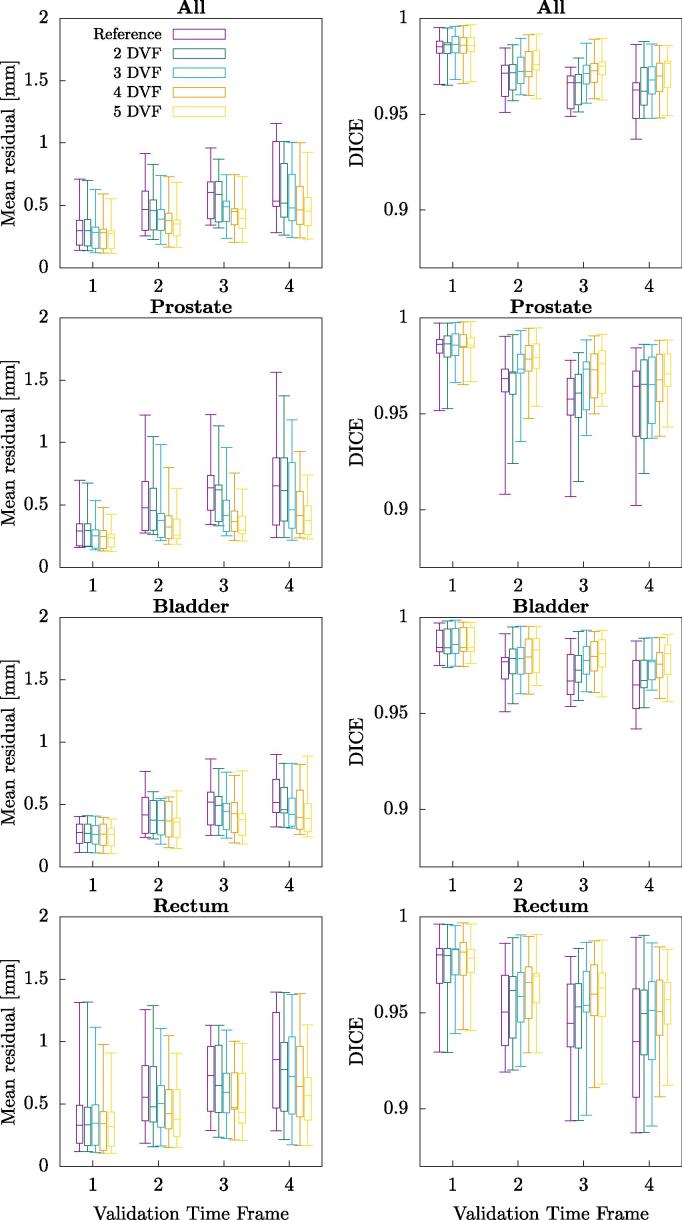
Boxplots of the mean residual displacements and DICE coefficient for all of the subjects for the last four, validation, time frames. Purple represents the value with no model applied. The green, blue, orange and yellow lines represents results for the models constructed with 2, 3, 4, and 5 displacement vector fields (DVFs), respectively. Whiskers are the maximum and minimum values. For each model all components have been utilized. This has been evaluated on the joint segmentation as well as on each of the included organs separately.

Models constructed with different amount of training data, ranging from two up to five DVFs and evaluated on the validation data, including all model principal components, shows that the addition of more data improved the accuracy of the model in terms of both mean residual and DICE (Fig. 4).

**Fig. 5:**
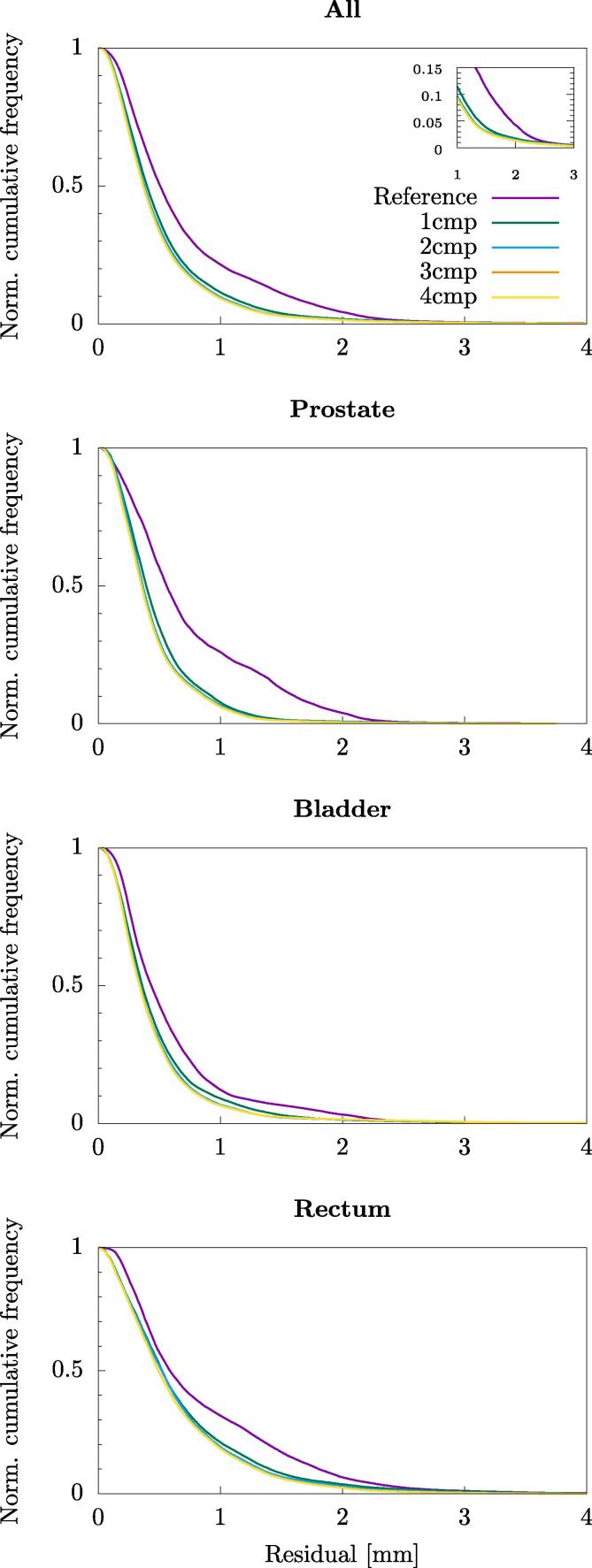
Normalized cumulative frequency distributions for the residuals. Summation from right to left, such that each point indicate the volume fraction moving at least the given distance. Purple line represent the results when there is no model applied and green, blue, orange and yellow the results when 1, 2, 3 and 4 principal components (cmp) have been utilized. The model has here been constructed with the full training set. The zoomed in window is around the largest voxel dimension.

In Fig. 5 the results on the validation data for different number of utilized components, for a model based on all five DVFs of the training data, is shown. For the joint results, i.e. all segmentations together, 21.4% of the voxels in the validation data has moved more than 1 mm which is reduced to 11.4%, 9.6%, 9.1% and 9.1% by utilizing the model with 1, 2, 3 and 4 principal components, respectively.

**Fig. 6:**
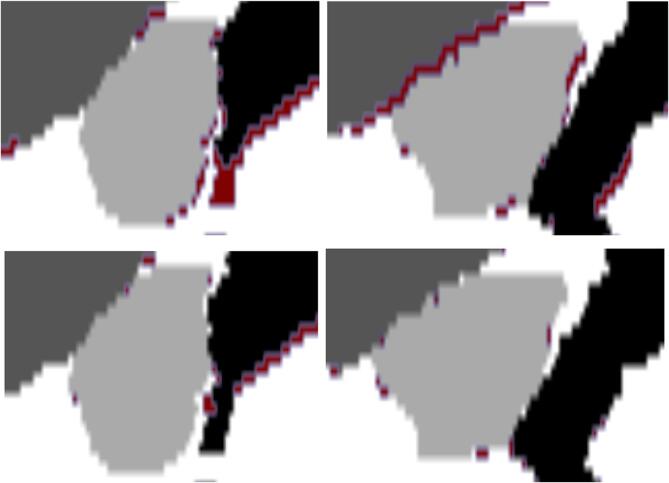
Example of anatomically located motion which is caught by the model, centered around the prostate and showing the intersection between the prostate(light grey), bladder(medium grey) and rectum(black), respectively, for two subjects. Red color indicates the state the anatomy has been moving to. The static, non-model, anatomy is shown in the top and the moved, model, anatomy in the bottom. The resolution is 2x1 mm in up–down and left–right direction respectively.

Fig. 6 shows the motion in a sagittal slice centered around the prostate and the corresponding improvement of overlap by utilizing a model of two representative subjects.

## 4 Discussion

In this work we constructed individual PCA based intrafractional motion models of the male pelvis based on dynamic scans of healthy volunteers. Through deformable image registration and PCA motion models were obtained, which were validated against the last time frames of the scans. Results showed that model accuracy seems to converge with one or two PCs, explaining the motion down to 1 mm.

The registration algorithm utilized in this work produced results with high accuracy and stability for all the time frames, crucial for obtaining an accurate motion model. The direction and size of the movement varied in between subjects due to different severity of bladder and rectum filling during the scan time as well as some muscle relaxation also taking place, consistent with e.g. [17]. The motion models of the different individuals could have possibly been more coherent with a consistent preparatory protocol, as indicated by e.g. [18] and [19] showing correlation between the rectal filling and prostate intrafraction motion.

These different motion patterns inherently produced motion models which differed in size and direction of their components, as shown in Fig. 2. This is consistent with other results indicating that for the prostate the intrafractional motion differs between patients (e.g. [20] and [21]) as well as for bladder ([22]) However, the cumulative variance as seen in Fig. 3 is very similar, concluding that a certain number of components explained roughly an equal amount of motion for every subject. The model was based on the joint segmentation to produce only one model for each subject. Hence, the weight was optimized for this joint segmentation, not for individual organs. Results for the model, applied on the validation data, seen in Fig. 4 showed that there is always an improvement of the results through a reduction in the residuals as well as increased DICE overlap for the joint segmentation, as expected. However as seen in some occasions, e.g. for the rectum, the results showed no improvement or even a slight worsening since the model was constructed like this which could indicate a weak correlation between the intrafractional motion of the modelled organs. [23] showed a stronger correlation between the rectal filling on prostate motion than bladder filling, indicating splitting of the models is advisable.

In the splitting of the data between training and validation, the common approach of a leave-one-out test was not utilized. Although reasonable for periodic motion patterns such as for respiratory models, e.g. [24], [7], it is less suitable in absence of such periodicity in motion as for bladder filling and rectum motion in the time frame of a treatment fraction. Instead, we took timing considerations into account and evaluated the model on times frames after the model training data. Utilizing a leave-one-out test or a K-fold cross validation for non-periodic continuous motion such as bladder and rectum filling over these time ranges would potentially have resulted in models which too a higher degree could explain the validation data. This since it would result in a time-wise interpolation rather than an extrapolation where a validation frame closer in time to the model training data is potentially more likely to be accurately explained by the model. Our approach was also consistent with any application scenario where the model is constructed prior utilization.

Considering the amount of training data results shown in Fig. 4 indicate that the addition of more data improves the model, suggesting that not all motion is captured in the training data.

For the validation data the results in terms of the residuals seemed to converge to approximately 1 mm, meaning that all motion found by the registration was not explained by the model. This is similar to result found in e.g. [25] and [11], who constructed similar models for interfractional images. In [25] they also concluded that larger initial deformation resulted in a larger reduction by the model which also is seen here. At the time frames in the validation data directly following the training data, the deformation was rather small and the reduction as well, while stepping forward in time the deformation increased as well as the reduction. However, due to the relatively short total acquisition time and the inherent small motions the size of the motion vectors was for many voxels of subvoxel size. The improvement was seen generally for the higher motion vectors. The increase of DICE overlap followed the same pattern as for the residual displacement, showing higher accuracy for the more rigidly moving prostate with a slight decrease in accuracy for the rectum. In general, the DICE overlap was already quite high, especially for the bladder which is a quite large homogeneous organ and the expected relative improvement with this measure is low.

We here chose an upper limit of six time frames for the training data. Increasing this amount is likely to increase the accuracy of the model, however with an increasing scan time. Consideration should also be given to how the model is affected by different scanning patterns such as temporal resolution and anatomical coverage, i.e. field of view. The relatively slow drift, in comparison to e.g. respiratory motion, potentially sets a lower requirement on the temporal resolution, although a very sparse update may not capture quicker motion patterns such as passing rectal gas bubbles. Here the relatively low temporal resolution of about 1 min stems from the fact that we covered not only the prostate but the whole pelvic region, however with a different MR sequence type, e.g. as in [20], temporal resolution could have been improved without loosing anatomical coverage. The limited total scan duration of 10–15 min sets an upper limit on the evaluation of the model accuracy over time. Considering a potential MR-Linac scenario with a quite long treatment time of >30 min may require a prolonged validation time span. However, the closer in time such a model is constructed with respect to the usage the more reliable it may be considered, hence imaging during MR idle time while planning and plan review is performed is a potential feasible scenario.

Since the models were constructed only on the edge voxels they were limited to only apply to homogeneous dose coverage of target. Dose painting approaches as in [4] would require the tracking of all target voxels and hence a corresponding model. As for the data comprising of scans of healthy volunteers and not real patients [26] indicated that at least for the prostate there is no significant difference in motion between patients and healthy volunteers, making these results valid in a patient setting as well.

In earlier works, considerable effort has been put in evaluating either interpatient, population-based models (e.g [25], [11]) or intrapatient (e.g. [8]) motion patterns as well as periodic intrafractional motion (e.g. [12]). However, little effort has been put in evaluating accuracy and potential benefits of intrafractional motion modelling in anatomical areas where the motion is in lack of periodicity. Inevitably, such models are likely to require more data and a more in depth evaluation of their temporal degradation since the motion states are not reoccurring. Here we created patient-specific intrafractional motion models to simultaneously cover the organs of interest in the male pelvic region. We have shown that it may be feasible to explain the motion with a few, 1–2, principal components, for potential application in creating individualised margin recipes or motion tracking.

## Declaration of Competing Interest

The authors declare that they have no known competing financial interests or personal relationships that could have appeared to influence the work reported in this paper.
