# Supplementary material for: Intrafractional motion models based on principal components in Magnetic Resonance guided prostate radiotherapy
Source: Phys Imaging Radiat Oncol. 2021 Oct 4;20:17–22. doi: 10.1016/j.phro.2021.09.004 (PMC8502906; doi:10.1016/j.phro.2021.09.004)
Supplement: Supplementary data 1 [file mmc1.pdf]

| **Setting** | **Value** |
|---:|---:|
| $T_R$[ms] | 5069 |
| $T_E$[ms] | 103 |
| Flip Angle[degrees] | 90 |
| FOV [mm] | 432x432x140-576x576x140 |
| Voxelsize[mm] | 1x1x2 |
| Dynamics | 10 |
| Acq.Time[s] | 760-916 |

Table 1: MR image acquisition settings. Transversal slice direction, subject feet first supine position. FOV direction and voxelsize is presented as Left-Right x Anterior-Posterior x Head-Feet. The field of view, FOV, varied slightly between subject to cover the whole pelvis avoiding fold over artifacts. The scanning sequence was a multi-slice 2D turbo spin echo $T_2$-weighted radial blade acquisition with a 100% percent sampling (SNR equivalent to cartesian sampling) Both repetition time $T_R$ and echo time $T_E$ varied slightly (a few ms) between subjects since the shortest possible time was applied. The dynamics where imaged concurrently yielding a temporal resolution of slightly above 1 min and with some variation between subjects.
